# SWEEPS-Assisted Antibacterial Photodynamic Therapy Against Dual-Species Biofilms in Mandibular Molars: An In Vitro Study

**DOI:** 10.3390/ph18040558

**Published:** 2025-04-10

**Authors:** Pargol Guity, Shima Afrasiabi, Ali Shahi Ardakani, Stefano Benedicenti, Antonio Signore, Nasim Chiniforush, Kiumars Nazari Moghaddam

**Affiliations:** 1 Department of Endodontics, Dental School, Shahed University, Tehran 3319118651, Iran; gpargol@yahoo.com; 2 Laser Research Center of Dentistry, Dentistry Research Institute, Tehran University of Medical Sciences, Tehran 1417614411, Iran; shafrasiabi@sina.tums.ac.ir (S.A.); shahi.ali1377@gmail.com (A.S.A.); 3 Department of Surgical Sciences and Integrated Diagnostics, University of Genoa, Viale Benedetto XV, 6, 16132 Genoa, Italy; benedicenti@unige.it; 4 Therapeutic Dentistry Department, Institute of Dentistry, I.M. Sechenov First Moscow State Medical University, Trubetskaya Str. 8, b. 2, 119992 Moscow, Russia; dr.signore@icloud.com; 5 Dentofacial Deformities Research Center, Research Institute for Dental Sciences, Shahid Beheshti University of Medical Sciences, Tehran 1983963113, Iran

**Keywords:** antimicrobial photodynamic therapy, dual-species biofilms, mandibular molars, natural photosensitizer, root canal therapy, SWEEPS, tooth color change

## Abstract

**Objectives**: The synergistic effect of shock wave-enhanced emission photoacoustic streaming (SWEEPS) and antimicrobial photodynamic therapy (aPDT) in mandibular molar root canal disinfection remains underexplored, particularly against dual-species biofilms that better simulate clinical conditions. This study evaluates their combined antimicrobial efficacy against *Enterococcus faecalis* and *Candida albicans* biofilms and assesses potential tooth discoloration caused by riboflavin and nano-curcumin. **Materials and Methods**: The mesiobuccal canals of 57 extracted mandibular molars were inoculated with *E. faecalis* and *C. albicans* biofilms. The antimicrobial effects were assessed using riboflavin or nano-curcumin with a 450 nm diode laser (BDL), SWEEPS, or their combinations, compared to 5.25% NaOCl (positive control) and saline (negative control). Biofilm reduction was quantified by colony-forming units (CFUs/mL), and discoloration was evaluated using the ΔE metric in the CIE L*a*b* color space. **Results**: Both microorganisms showed a significant decrease in colony numbers in all experimental groups compared to the negative control (*p* < 0.001), except for *E. faecalis*, where no significant difference was observed between the riboflavin/nano-curcumin groups and the negative control. Combining riboflavin or nano-curcumin with SWEEPS or BDL significantly enhanced antimicrobial efficacy compared to individual treatments (*p* < 0.001). The combined photodynamic therapy and SWEEPS groups showed the lowest colony counts. The ΔE values were, on average, 1.81 for riboflavin and 1.09 for nano-curcumin. **Conclusions**: The combination of SWEEPS and aPDT effectively reduces *E. faecalis* and *C. albicans* biofilms in molars, supporting its potential as an adjunct in endodontic disinfection. Minimal discoloration further highlights its clinical applicability.

## 1. Introduction

Apical periodontitis, characterized by its microbial origin, is a widespread inflammatory disease impacting human health [1]. Both bacteria and fungi have been identified in association with this condition [2]. Persistent signs and symptoms in treated teeth indicate apical periodontitis, which may have persisted or returned, referred to as post-treatment apical periodontitis [3]. This condition is believed to arise from a microbial community rather than a single pathogen [4,5]. *E. faecalis* is frequently detected in root canals of teeth affected by apical periodontitis after treatment and is more common in post-treatment cases, likely due to its tolerance to unfavorable conditions [6]. While bacteria dominate endodontic infections, other species, such as *Candida* spp., especially *C. albicans*, are found in approximately 18% of post-treatment cases, possessing virulence factors that may contribute to endodontic pathologies [2,7,8]. Therefore, effective disinfection strategies are essential for enhancing infection control and improving treatment outcomes.

Mesial canals of mandibular molars are often studied due to their anatomical complexity, including isthmuses [9,10]. This complexity is a significant factor contributing to the failure of root canal treatment, as it complicates adequate disinfection and increases the likelihood of procedural errors. Such errors lead to a higher prevalence of periapical lesions [11]. Complications in Vertucci type IV canals can heighten the risk of treatment failure due to increased bacterial proliferation potential, and studies indicate that the mesial canals of mandibular molars generally correspond to this type [12]. Consequently, effective disinfection of these canals poses a major challenge.

Enhancing chemical disinfection methods is a logical approach, as mechanical methods have proven inefficient. Despite new filing systems, many leave unprepared walls in the canal, compromising treatment success [13,14]. This issue may arise from a lack of compatibility between available instruments and apical geometries [15]. Recently, there has been a shift toward greater reliance on chemical disinfection methods, moving away from traditional mechanical approaches [16].

Sodium hypochlorite (NaOCl) is the most commonly used canal irrigant due to its strong antimicrobial properties, ability to dissolve organic tissue, lubricating effect, and cost-effectiveness [16]. However, if NaOCl extrudes beyond the apex, it can damage periapical tissues [17]. This has prompted research into effective, safe, and potentially natural alternatives [18]. The delivery of irrigants to complex canal systems is crucial, as conventional syringe methods do not achieve the desired antimicrobial efficacy, emphasizing the need for activation methods [16]. Ultrasonically activated irrigation (UAI) is a common activation method, but it raises the risk of file fractures or dentin erosion [19,20,21].

Lasers are widely used in dentistry, including for laser-activated irrigation (LAI) in endodontics. Studies suggest that LAI has better antimicrobial efficacy compared to UAI [22,23,24]. Erbium-doped yttrium aluminium garnet (Er:YAG) lasers emit light that is strongly absorbed by water. This creates cavitation bubbles that generate turbulence and aid the removal of debris without direct contact with the canal [22,25]. Shock wave-enhanced emission photoacoustic streaming (SWEEPS), a newly introduced mode in Er:YAG laser systems, further enhances this process by using synchronized laser pulses. The first pulse generates a cavitation bubble that begins to collapse, while the second pulse accelerates this collapse and creates shock waves. These waves, together with the secondary cavitation bubbles near the canal walls, create shear forces that improve debris removal and smear layer disruption [26]. In addition to its improved cleaning performance, SWEEPS also has a favorable safety profile. A major concern in irrigant activation is apical extrusion, which can cause periapical irritation and postoperative pain [27]. A clinical study has shown that SWEEPS leads to the least postoperative pain compared to other activation methods [28]. In addition, the shock waves penetrate deep into the lateral canals and microscopic tubules and improve disinfection [22]. SWEEPS is also effective in the cleaning of curved root canals [29] and removing residual sealer during retreatment [30], which emphasizes its versatility in endodontic practice.

Antimicrobial photodynamic therapy (aPDT) is an approach in which a photosensitizer is activated by radiation of a specific wavelength, leading to the generation of reactive oxygen species (ROS) that destroy the target cells [31]. The success of aPDT depends on photosensitizers that demonstrate high quantum efficiency [32]. Recently, there has been growing interest in natural photosensitizers, such as curcumin from turmeric, due to benefits such as lower production costs and environmental friendliness [33]. However, due to the low water solubility of curcumin and its poor bioavailability, nanotechnology has been used to improve its properties. One such advance is a nanomicelle formulation containing curcumin (nano-curcumin), which not only improves these properties but also has lower toxicity to normal cells than the conventional form [34,35]. Curcumin has absorption maxima between 420 and 480 nm [36]. This improved form is increasingly being used in aPDT, with promising results emerging [37]. Riboflavin (vitamin B_2_), another natural photosensitizer, is biocompatible and also generates ROS [38]. Riboflavin is mainly found in leafy vegetables, but can also be obtained from mushrooms and dairy products. It is considered a promising photosensitizer, capable of generating ROS when exposed to blue light [39,40]. While recent studies on aPDT indicate promising results in improving root canal disinfection, more research is needed to further validate these results [31].

The use of photosensitizing agents in aPDT introduces the potential risk of tooth discoloration after their application. It is essential to carefully consider this possibility and assess the degree of discoloration resulting from their use. Color measurement relies on colorimetry, which uses digital methods to quantify the color perceived from an object. To analyze color variations, the Munsell and CIE L*a*b* (Commission Internationale de l’Eclairage L*a*b*) systems are widely applied, with the latter being endorsed by the American Dental Association (ADA). The CIE L*a*b* model represents all natural colors as mixtures of three primary colors—red, green, and blue—in defined ratios [41,42,43].

Given the ongoing challenge of achieving optimal antimicrobial efficacy, against biofilms during canal cleaning in endodontics, especially in anatomically complex teeth, the present study aimed to evaluate the antimicrobial efficacy of aPDT in combination with the SWEEPS technique against dual biofilms of *E. faecalis* and *C. albicans*. Additionally, considering the use of photosensitizers, the potential for tooth discoloration as a result of their application was also investigated.

## 2. Results

### 2.1. Intracanal Biofilm Formation

**Figure 1:**
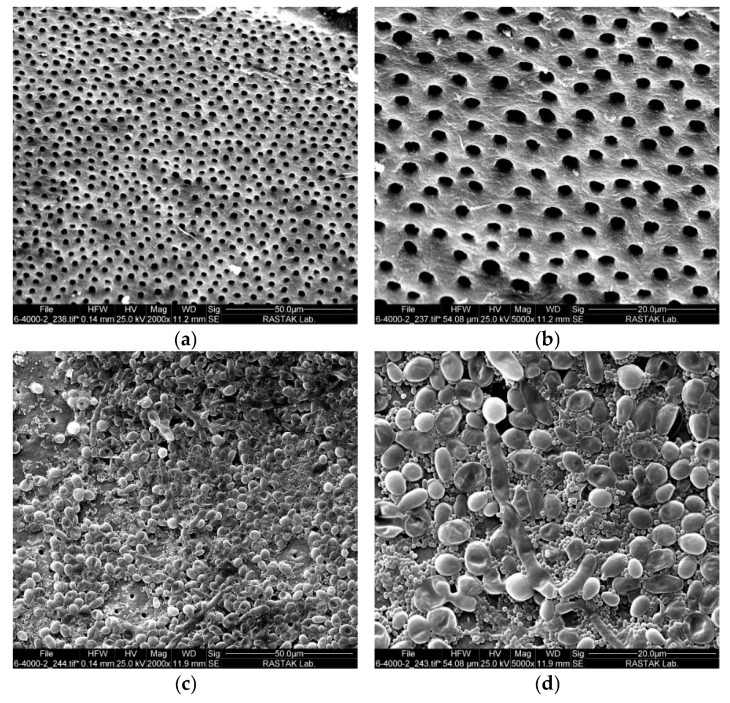
FESEM micrographs illustrating the root canal surface and biofilm formation: (**a**) root canal surface devoid of the smear layer, revealing open dentinal tubules at a magnification of 2000×; (**b**) the same area at a higher magnification of 5000×; (**c**) overview of the thick biofilm structure formed by *E. faecalis* and *C. albicans* at a magnification of 2000×; and (**d**) distinct visualization of microbial structures at a magnification of 5000×.

In the field emission scanning electron microscopy (FESEM) images of the negative control sample, it is evident that the smear layer was entirely eliminated, allowing for a clear view of the root surface and open root tubules (Figure 1a and 1b at magnifications of 2000× and 5000×, respectively). A biofilm consisting of microorganisms developed on the surface of the root canal and is clearly identifiable in the FESEM images. Figure 1c provides an overview of the thick biofilm structure at a magnification of 2000×. Upon closer inspection, as shown in Figure 1d, distinct visualization of *E. faecalis* and *C. albicans* is achieved at higher magnifications.

### 2.2. Antimicrobial Efficacy of aPDT

**Figure 2:**
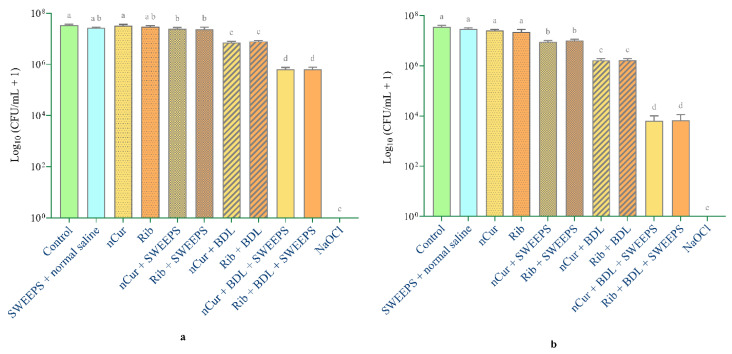
Impact of various experimental groups on the survival of *E. faecalis* (**a**) and *C. albicans* (**b**) cells. The letters above each group indicate the homogeneous subsets identified through statistical analysis. Rib: riboflavin, nCur: nano-curcumin, SWEEPS: shock wave-enhanced emission photoacoustic streaming, BDL: blue diode laser, NaOCl: 5.25% sodium hypochlorite.

Figure 2 presents a detailed illustration of the mean values and standard deviations across the different study groups, with Figure 2a showing the survival rate of *E. faecalis* and Figure 2b depicting the survival rate of *C. albicans*. A notable distinction between the groups was verified through ANOVA analysis, with a statistical significance level of *p* < 0.001. The outcomes of Tukey’s multiple comparisons test are presented as the letters of significant difference in Figure 2.

Colony numbers significantly decreased in most experimental groups compared to the negative control (*p* < 0.001), particularly in those combining riboflavin or nano-curcumin with SWEEPS, blue diode laser (BDL), or both. However, no significant difference was observed in the riboflavin (*p* = 0.808), nano-curcumin (*p* > 0.999), and SWEEPS + normal saline (*p* = 0.061) groups for *E. faecalis*, as well as in the riboflavin (*p* = 0.105), nano-curcumin (*p* = 0.524), and SWEEPS + normal saline (*p* = 0.957) groups for *C. albicans* compared to the negative control.

Notably, no microbial presence was observed in the NaOCl group.

For both microorganisms, treatments combining riboflavin or nano-curcumin with either SWEEPS, BDL, or both were significantly more effective than their use alone (*p* < 0.001), except for *E. faecalis*, where no statistically significant difference was observed between the riboflavin and riboflavin + SWEEPS groups (*p* = 0.095).

As shown in Figure 2, both the riboflavin + BDL and nano-curcumin + BDL groups showed a significantly greater reduction in colony numbers for both microorganisms compared to the riboflavin + SWEEPS and nano-curcumin + SWEEPS groups (*p* < 0.001).

The colony counts in the combined photodynamic therapy and SWEEPS groups (riboflavin + BDL + SWEEPS and nano-curcumin + BDL + SWEEPS) were significantly lower compared to their individual counterparts (riboflavin + BDL, riboflavin + SWEEPS, nano-curcumin + BDL, and nano-curcumin + SWEEPS) for both microorganisms (*p* < 0.001).

### 2.3. Outcomes of Tooth Discoloration

The values of L*, a*, b*, and ΔE for each specimen are presented in Table 1.

## 3. Discussion

Failed root canal treatments are nine times more likely to harbor *E. faecalis* compared to primary endodontic infections. Its prevalence in root-filled teeth with periradicular lesions has been reported to range from 24% to 77%. *E. faecalis* exhibits various virulence factors, including adherence to host cells, expression of competitive proteins, and modulation of host immune responses Additionally, *E. faecalis* forms biofilms, which enhance its resistance to phagocytosis, antibodies, and antimicrobials by up to 1000 times compared to non-biofilm producing organisms. These characteristics enable *E. faecalis* to overcome the challenges of survival within the root canal system and contribute to its persistence in endodontic infections [44,45].

*C. albicans* strains isolated from the root canal exhibit high proteolytic activity and may suggest a pathogenic role for it in endodontic infections [46]. These yeasts can adapt to a variety of environmental conditions and adhere to many surfaces, including dentin and root filling materials. Moreover, *C. albicans* can produce hydrolytic enzymes, undergo morphologic transition, form biofilm, and evade and modulate the host defense [47]. The ability of *Candida* spp. to strongly adhere to dentin, forming germ tubes, hyphae, and a thick extracellular matrix, contributes to increased resistance against the immune system response, and therefore it may contribute to the pathogenesis of periradicular diseases and also to chemical agents [48]. *E. faecalis* showed more resistance to starvation in the presence of *C. albicans*, which might provide some essential ingredients for *E. faecalis* survival [49,50,51]. Moreover, an in vitro study has indicated that *E. faecalis* promotes the hyphal morphogenesis and biofilm formation of *C. albicans* [52].

Root preparation has two main objectives: the removal of infected tissue and the creation of sufficient space for obturation. Achieving these goals is a challenge both at the macroanatomical level (curvature and calcifications) and at the microanatomical level (the isthmus, lateral canals, and apical delta) [53]. Significant progress has been made to overcome the macroanatomical challenges in mechanical root preparation, particularly with the introduction of rotary files with special properties [54]. Even with the use of endodontic instruments with different designs, kinematics, and heat treatments, there is no documented system in the literature that can fully reach all surfaces of the root canal [55]. However, using a technique without instrumentation offers the advantage of preserving the tooth structure and reducing the risks associated with the instrumentation of curved and narrowed root canals [56]. The microanatomical structures within the root canal system pose a challenge for mechanical cleaning, as there are inaccessible areas. In particular, areas such as the isthmus, which is frequently observed in the mesial canals of mandibular molars, serve as a reservoir for biofilm and provide protection against disinfection processes [23,57]. The physical limitations of the instruments and the short duration of irrigant contact during canal preparation result in inadequately prepared walls in areas such as the isthmus, lateral canals, and lateral branches [58]. Studies have shown that syringe irrigation has an acceptable efficacy for disinfection and biofilm removal in single-rooted teeth with uncomplicated anatomy. However, in more complicated tooth morphologies, its capacity is limited to the main canal. In these cases, techniques involving irrigant activation or additional methods are used to achieve the biological goal of endodontics, which is to maximize the reduction of microorganisms [16].

NaOCl effectively reduces endodontic infections during root canal treatment [59], but it has notable drawbacks. It can damage tissues, alter dentin’s mechanical properties, and weaken bond strength due to its proteolytic activity [60,61,62,63]. Its efficacy is concentration-dependent, yet higher concentrations increase the risk of apical extrusion and postoperative pain, potentially leading to severe complications, such as necrosis, swelling, or neurological damage. Additionally, NaOCl has an unpleasant taste and odor, can irritate oral mucosa, and may cause ocular and skin injuries [64].

UAI is likely the most widely used adjunct method for enhancing the efficacy of irrigation [65]. The motion of ultrasonic files induces acoustic streaming within the intracanal solution, subsequently enhancing mechanical cleaning by augmenting shear stress on the canal walls. UAI has been shown to cause uncontrolled dentin removal in both straight and curved root canals [21]. Another limitation of using UAI is the requirement to position the tip within 2–3 mm of the working length, which is a challenge in curved and unprepared canals [66].

To enhance canal irrigation, several additional methods have been introduced. One of these methods is LAI, which utilizes the phenomenon of cavitation to enhance the potency of the irrigant in removing debris. This technique leverages the rapid formation and collapse of vapor bubbles induced by laser energy in pulsed mode. The laser utilized in LAI is the erbium laser, whose light exhibits optimal absorption in aqueous solutions [67]. This property leads to overheating and boiling of the irrigant, resulting in the generation of vapor. When the temperature rises, these vapor bubbles expand and then quickly collapse (implosion). The mechanical effects of cavitation contribute to the effective removal of debris and microbial biofilms from the canal walls, enhancing the overall cleaning and disinfection process. This modality of LAI is called Photon-Induced Photoacoustic Streaming (PIPS). To enhance the technique, another modality known as SWEEPS has been introduced, which uses double pulsing to create shock waves. The erbium tip is positioned in the pulp chamber, resulting in vapor formation around it. The primary bubble undergoes coronal-directed implosion. This coronal implosion generates negative pressure within the canal, which may effectively mitigate vapor lock [22]. The vapor lock, which is characterized by the generation of gas bubbles, poses a challenge for effective cleaning in the apical third of the root canal [68]. Another potential advantage of generating negative pressure is the reduction of apical extrusion of the irrigant, resulting in less toxic effects and less postoperative pain. Erbium laser-induced cavitation bubbles in the pulp chamber or at the canal entrance lead to substantial movement of the irrigant through the underlying canal space. This is in contrast to other irrigation techniques, such as UAI, where the tip must be placed intracanalicularly. The ability to perform the procedure without the need for precise intracanal positioning is believed to simplify the process and reduce operator dependency [22].

Another complementary irrigation method is aPDT, a minimally invasive approach that induces microbial cell death by generating free radicals. Two natural photosensitizers, nano-curcumin and riboflavin, are used for this purpose. Riboflavin, unlike NaOCl, has a positive effect on adhesion strength [69,70], and nano-curcumin has a minimal effect on mechanical properties [71].

In this study, we used the mesiobuccal canal of a mandibular molar, which presents anatomical complexity, and employed a combination of SWEEPS and aPDT. Considering the interactions between microorganisms within biofilms, this study specifically aimed to assess the efficacy of this approach against mixed-species biofilms. Additionally, natural photosensitizers were utilized. This approach was based on the initial hypothesis that SWEEPS would improve the distribution of the photosensitizer throughout the canal, thereby increasing antimicrobial efficacy. The results indicated that the combined application of aPDT and SWEEPS demonstrated notable antimicrobial efficacy for both photosensitizers, resulting in an approximately 3.7 Log_10_ colony-forming units (CFUs/mL) reduction for *C. albicans* and a 1.7 Log_10_ CFU/mL reduction for *E. faecalis*. It is also important to highlight that the antimicrobial performance of the aPDT groups was superior to that of the SWEEPS groups alone. It is necessary to address the existing literature on aPDT in root canal disinfection to better understand the significance of the present findings. Moradi et al. [72] investigated the effect of aPDT with curcumin and riboflavin on *E. faecalis* biofilms in a tooth model. Their results showed that both photosensitizers, when activated with a Light-Emitting Diode (LED), significantly reduced the number of bacteria. These results are consistent with the present study. However, in contrast to the current study, Moradi et al. used a single-species biofilm and single-rooted teeth. The results of this study are also consistent with previous studies on the synergistic effect of SWEEPS and aPDT in root canal disinfection. SWEEPS has been shown to improve the antimicrobial efficacy of aPDT using indocyanine green (ICG) and methylene blue (MB), leading to significant biofilm reduction [73,74]. It is important to note that ICG has photothermal effects that may raise the intracanal temperature above the physiological threshold of the periodontal ligament, which could pose a risk to the surrounding tissues. Furthermore, MB has also been associated with the risk of tooth discoloration. A study by Costa et al. evaluated the potential for tooth discoloration following the application of MB. They found an average ΔE value of >8, indicating a significant color change [75]. These studies also focused on single-rooted teeth and *E. faecalis* biofilms.

The potential for tooth discoloration associated with the use of dyes in dental treatments was examined by assessing the effects of riboflavin and nano-curcumin under simulated clinical conditions during root canal therapy. Unlike conventional methods that rely on sample immersion, this study replicated clinical scenarios to evaluate color changes in the tooth crown. The results demonstrated that the ΔE values for both riboflavin and nano-curcumin were below the clinically perceptible threshold of 3.7, indicating minimal risk of visible discoloration [76,77,78]. To contextualize these findings within the literature, a systematic review conducted in 2023 on tooth discoloration following photodynamic therapy in endodontics reported that among various dyes, including MB, toluidine blue O, malachite green, ICG, and curcumin, only curcumin and ICG did not cause significant discoloration [79].

In this study, a mandibular molar dental model and dual-species biofilm were employed to closely replicate challenging clinical conditions. A live/dead assay to quantify the proportion of non-viable bacteria within the biofilm could have provided further insight into the results. In addition, future research should consider the use of confocal laser scanning microscopy for a more detailed assessment of biofilm structure. It is also recommended that further studies investigate the potential effects of nano-curcumin and riboflavin on periapical tissues.

## 4. Materials and Methods

### 4.1. Materials and Instruments

Materials: 37% phosphoric acid (Cobalt Etch 37%, Cobalt, Tehran, Iran), Adper Single Bond Universal (3M ESPE, St. Paul, MN, USA), resin composite (Filtek Z250, 3M ESPE, St. Paul, MN, USA), glass ionomer restorative material (Fuji II, GC, Tokyo, Japan), NaOCl solution (Cobacid, Cobalt, Tehran, Iran), EDTA solution (Morvabon, Tehran, Iran), paper cones (Meta Biomed, Chungcheongbuk-do, Republic of Korea), brain heart infusion (BHI) broth and agar (Ibresco, Tehran, Iran), Sabouraud dextrose (SD) broth and agar (Ibresco, Tehran, Iran), nano-curcumin (Exir Nano Sina, Tehran, Iran), riboflavin (Harman Finochem Ltd., Mumbai, India). *E. faecalis* (IBRC-M 11130) and *C. albicans* (ATCC 10231) were obtained from the National Center of Genetic and Biological Resources of Iran.

Instruments: microscope (Mediworks microscope, Beijing, China), dental RVG sensor (iSensor, Woodpecker, Guilin, China), sonic scaler device (UDS.k, Woodpecker, Guilin, China), microtome, LED (Vivadent Bluephase N Polywave, Ivoclar, Schaan, Liechtenstein), K-file (Mani, Takanezawa, Japan), RaCe rotary file (FKG, Lachaux-Fonds, Switzerland), FESEM (FESEM, FEI SEM QUANTA 200 EDAX EDS SILICON DRIFT 2017, Hillsborough, OR, USA), Er:YAG laser (LightWalker AT, Fotona, Ljubljana, Slovenia), tip of SWEEPS device (Sweeps600, Fotona, Ljubljana, Slovenia), BDL (Wiser 3, Doctor Smile, Brendola, Italy), tip of BDL device (Tip Endodontics 200 µm, Doctor Smile, Brendola, Italy), spectrophotometer (SpectroShade, MHT, Verona, Italy).

### 4.2. Sample Selection and Preparation

The study protocol was approved by the Ethics Committee of the School of Dentistry, Shahed University (IR.SHAHED.REC.1403.008). Fifty-seven permanent human mandibular molars with less than 25 degrees were selected. The curvature of the roots was assessed by the method of Schneider [80]. Teeth with a hopeless periodontal prognosis were selected, and the patients were informed about the purpose of the research. Teeth with severe structural damage, such as cracks, fractures, extensive caries, or open apical foramen, were excluded from the examination. All samples were examined microscopically at a magnification of 10× to identify any defects on the root surface. Mesial roots that exhibited a type IV Vertucci canal configuration were intentionally included, as determined by periapical radiographs in the mesiodistal and buccolingual directions. Teeth showing calcification or Vertucci type II were excluded from the study [81].

First, the outer surface of the tooth was cleaned with a sonic scaler device. In the next step, a microtome was used to separate the mesial root from the distal root, and the crown of the tooth was sectioned from the cemento-enamel junction, leaving 13 mm of the mesial root length intact. The defect in the distal wall of the mesial root was repaired using resin composite. To prevent composite materials from entering the canal, a polytetrafluoroethylene tape was used to block the root canal orifice. Then, 37% phosphoric acid was applied to the tooth surface for 15 s, rinsed for 10 s, and then dried with an air syringe for 10 s. Adper Single Bond Universal adhesive was then applied to the surface with a microbrush and carefully dried with an air spray. The second layer of bonding was applied and gently dried again, then light-cured with a LED for 20 s. The device operated in a wavelength range of 385 to 515 nm. It had a power density of 1200 mW/cm^2^, and the light guide was 10 mm in diameter. The resin composite was applied into the defect area and light-cured for 40 s. In addition to establishing patency and directly determining the working length (0.5 mm shorter than the length the file protruded beyond the apex) with a #10 K-file, separation of the apices of the mesial canals was ensured. A glass ionomer restorative material was used to cover the orifice of the mesiolingual canal. The mesiobuccal canal was shaped using the RaCe rotary file system in the following order: (#35/8%)–(#15/4%)–(#20/4%)–(#25/4%)–(#30/6%). During all preparation steps, the root canal was irrigated with a NaOCl solution using a side-vented irrigation syringe and a 27-gauge needle. In the last step, 17% EDTA was applied for 60 s, followed by a 30 s rinse with 5.25% NaOCl to remove the smear layer. The apexes of the teeth were sealed using a glass ionomer restorative material, while a layer of varnish was applied to the root surfaces to prevent microbial contamination. The root canals of the teeth were dried using paper cones and then sterilized.

### 4.3. Root Canal Contamination

Bacterial cultures were grown overnight in BHI broth under aerobic conditions at 37 °C, while fungal culture was conducted in SD broth. The microbial suspension was prepared to achieve a concentration of 0.5 McFarland (1.5 × 10^8^ CFU/mL). To initiate biofilm formation, *E. faecalis* and *C. albicans* were inoculated with a concentration of 10^7^ and 10^5^ CFU/mL, respectively, and then incubated for 10 days at 37 °C under aerobic conditions. During the incubation period, 10 μL of fresh microbial suspension was added every 2 days.

### 4.4. Control of Intracanal Biofilm Formation

After the above-mentioned period, two samples were selected to check the biofilm formation on the canal walls using field emission scanning electron microscopy. Furthermore, a control sample without microbial contamination was used to assess the effectiveness of the smear layer removal procedures. The roots were longitudinally sectioned using a chisel and a hammer [82]. The samples were rinsed with phosphate-buffered saline (PBS). To fix the samples, they were soaked in 2.5% glutaraldehyde for 30 min and then rinsed with PBS for 10 min. They were then post-fixed in a 1% (*w*/*v*) osmium tetroxide solution for 20 min and washed with PBS as a final step. Dehydration was achieved by gradually increasing the ethanol concentrations. Each concentration was maintained for 5 min. The samples were affixed to a base and then coated with gold palladium. They were then examined under a FESEM at magnifications of 2000× and 5000×.

### 4.5. Light Sources and Photosensitizers

The SWEEPS action was accomplished using an Er:YAG laser with a wavelength of 2940 nm operating at parameters of 0.3 W average power, 15 Hz frequency, and 20 mJ per pulse. This was executed in an ultrashort, free-running pulse mode with a duration of 25 μs. The device tip was placed at the canal orifice and activated for a total duration of 60 s. This consisted of 30 s of activation followed by a 30 s resting period, and then another 30 s of activation [74]. A BDL operating at a wavelength of 450 nm and an output power of 200 mW was used for 30 s [83]. The tip of the device was positioned at the apical third of the canal, 2 mm away from the working length. Commercial nano-curcumin at a concentration of 5 mg/mL was prepared in a 0.9% sodium chloride solution. The curcumin encapsulation efficiency is close to 100%, with a mean particle diameter of 9.5 ± 0.1 nm [34]. Riboflavin at a concentration of 40 mg/mL was also prepared in a 0.9% sodium chloride solution.

### 4.6. Experimental Design

**Figure 3:**
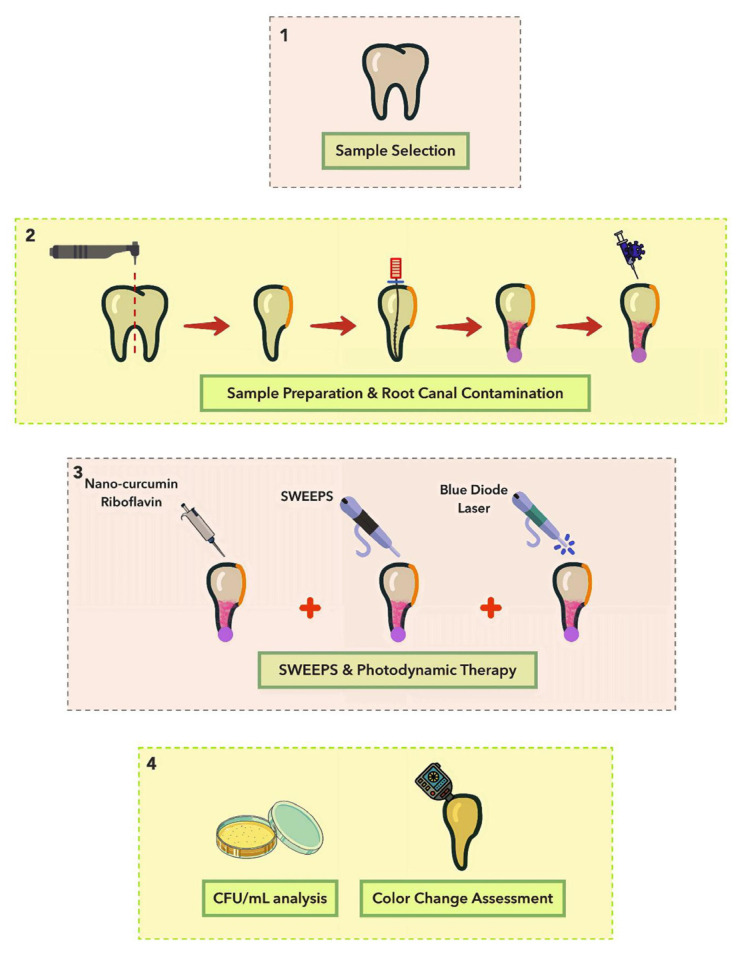
A schematic illustration of the different phases of the study.

The chosen teeth were allocated randomly into 11 groups (*n* = 5):

1. Normal saline (negative control): 10 μL of normal saline was injected into the canal using a micropipette, with samples kept in the dark for 5 min [74].
2. NaOCl (positive control): 10 μL of 5.25% NaOCl was injected using a micropipette, with samples kept in the dark for 5 min.
3. Normal saline + SWEEPS: the normal saline was applied like group 1 and then the SWEEPS technique was performed.
4. Nano-curcumin: 10 μL of nano-curcumin was injected into the canal using a micropipette, with samples kept in the dark for 5 min.
5. Riboflavin: 10 μL of riboflavin was injected into the canal using a micropipette, with samples kept in the dark for 5 min.
6. Nano-curcumin + SWEEPS: the root canal was filled by nano-curcumin (like group 5). Then, the SWEEPS technique was performed.
7. Riboflavin + SWEEPS: the root canal was filled by riboflavin (like group 6). Then, the SWEEPS technique was performed.
8. Nano-curcumin + BDL: the root canal was filled by nano-curcumin (like group 5). Then, according to the specified parameters, the BDL was irradiated.
9. Riboflavin + BDL: the root canal was filled by riboflavin (like group 6). Then, according to the specified parameters, the BDL was irradiated.
10. Nano-curcumin + BDL + SWEEPS: the application of nano-curcumin and SWEEPS technique was conducted like group 7 and then, the BDL was irradiated.
11. Riboflavin + BDL + SWEEPS: the application of riboflavin and SWEEPS technique was performed like group 8, and then the BDL was irradiated. A schematic illustration of the different phases of the study is shown in Figure 3.

### 4.7. Assessment of Antimicrobial Efficacy

Following the therapeutic procedure, the root canals of the teeth were dried using a paper cone. Following this, each sample was placed into microtubes containing 1 mL of PBS. The biofilm was then separated by vortexing the microtube for 60 s. Afterwards, 10 μL of the suspension was subjected to a series of dilutions (10 dilutions). Subsequently, 10 μL from each dilution was cultured on BHI agar supplemented with amphotericin B for *E. faecalis* and SD agar supplemented with chloramphenicol for *C. albicans*. The plates were then incubated at 37 °C for 24 h. The number of microbial colonies was counted [84].

### 4.8. Assessment of Color Change

Ten human adult teeth (mandibular premolars) with intact crowns and roots, free from cracks, caries, or other defects, were selected. These teeth had been extracted for periodontal or orthodontic reasons. Notably, single-rooted mandibular premolars were selected in this section to assess the impact of riboflavin and nano-curcumin on crown discoloration. This selection was intentional, as maintaining the structural integrity of the crown was essential to avoid the need for composite restoration. Root canal preparation was performed according to the protocol described in Section 4.2. The teeth were randomly assigned to two groups (*n* = 5): riboflavin and nano-curcumin.

The baseline color of the buccal surface of each crown was measured using a spectrophotometer. Following this, 10 μL of riboflavin was injected into the root canals of the first group, while 10 μL of nano-curcumin was injected into the second group. After a 5 min incubation period, the solutions were aspirated from the canals. Subsequently, the root canals and pulp chambers were rinsed with normal saline and dried using paper cones. The buccal surface color of each sample was then reassessed [85].

Color data were recorded based on the CIE Lab* system, where L* represents lightness, a* indicates the red-green axis, and b* denotes the yellow-blue axis. The color change (ΔE) was calculated using the following formula [86]:

$$\Delta E=\sqrt{[{\left(\Delta L\right)}^{2}+{\left(\Delta a\right)}^{2}+{\left(\Delta b\right)}^{2}}$$

where:

ΔL = L1 − L0, Δa = a1 − a0, and Δb = b1 − b0

### 4.9. Statistical Analysis

The conformity of CFU/mL data to a normal distribution was verified through a Kolmogorov–Smirnov test, with a significance level established at *p* < 0.05. A one-way ANOVA followed by Tukey’s test was used to compare the bacterial CFU/mL among the various groups. GraphPad Prism version 10.0.2 (GraphPad Software, Boston, MA, USA) was used for the statistical analysis. The significance threshold was set at *p* < 0.05.

## 5. Conclusions

Overall, this study highlights the promising potential of aPDT using riboflavin and nano-curcumin in combination with the SWEEPS technique to improve antimicrobial efficacy against resistant microorganisms in endodontic treatments. This approach could reduce the risk of persistent infections, enhancing long-term treatment success. In addition, the low ΔE values observed for both agents suggest minimal aesthetic compromise, making this method a suitable option for maintaining tooth appearance. These findings support the clinical value of this combined therapy as an adjunct to conventional treatments, though further clinical trials are needed to confirm its long-term efficacy and safety.

## Figures and Tables

**Table 1:** L*, a*, and b* values and ΔE for each sample.

Sample		Before Treatment	After Treatment	ΔE
Rib 1	L	74.92 ± 1.78	75.48 ± 0.86	1.39 ± 0.67
	a	1.98 ± 0.42	2.34 ± 0.34	
	b	17.38 ± 1.70	18.86 ± 1.52	
Rib 2	L	75.30 ± 1.06	77.10 ± 2.05	1.93 ± 0.99
	a	0.10 ± 0.02	0.40 ± 0.04	
	b	19.00 ± 0.81	21.30 ± 1.80	
Rib 3	L	76.90 ± 0.67	75.20 ± 1.45	2.07 ± 0.79
	a	0.70 ± 0.12	0.90 ± 0.25	
	b	19.80 ± 1.87	25.70 ± 1.05	
Rib 4	L	73.50 ± 1.34	75.30 ± 1.43	1.80 ± 0.14
	a	2.70 ± 0.83	2.90 ± 0.26	
	b	13.50 ± 0.74	13.25 ± 0.92	
Rib 5	L	72.30 ± 0.92	73.80 ± 1.44	1.85 ± 0.52
	a	2.70 ± 0.26	2.10 ± 0.51	
	b	16.30 ± 1.21	18.10 ± 1.48	
nCur 1	L	77.30 ± 1.38	77.10 ± 1.01	0.78 ± 0.37
	a	0.10 ± 0.05	0.60 ± 0.03	
	b	17.10 ± 0.38	18.60 ± 0.79	
nCur 2	L	77.30 ± 0.84	77.00 ± 0.65	0.37 ± 0.19
	a	0.90 ± 0.12	1.20 ± 0.08	
	b	17.50 ± 1.10	18.20 ± 1.08	
nCur 3	L	74.70 ± 1.76	76.40 ± 1.41	1.74 ± 0.35
	a	2.20 ± 0.09	2.40 ± 0.05	
	b	16.50 ± 1.60	14.70 ± 1.03	
nCur 4	L	74.30 ± 1.19	74.40 ± 1.34	0.63 ± 0.16
	a	2.80 ± 0.44	2.50 ± 0.65	
	b	17.10 ± 1.07	15.30 ± 0.88	
nCur 5	L	74.00 ± 1.84	75.90 ± 0.99	1.92 ± 0.86
	a	2.50 ± 0.45	2.00 ± 0.24	
	b	13.70 ± 0.68	14.2 ± 1.32	

Rib: riboflavin, nCur: nano-curcumin.

## Data Availability

The original contributions presented in this study are included in the article. Further inquiries can be directed to the corresponding authors.
